# Correction: Impact of physical structure of granular sludge on methanogenesis and methanogenic community structure

**DOI:** 10.1039/c9ra90074h

**Published:** 2019-10-15

**Authors:** Xiaofang Pan, Lina Wang, Nan Lv, Jing Ning, Mingdian Zhou, Tao Wang, Chunxing Li, Gefu Zhu

**Affiliations:** Key Laboratory of Urban Pollutant Conversion, Institute of Urban Environment, Chinese Academy of Sciences Xiamen 361021 China gfzhu@iue.ac.cn +86-592-6190790 +86-592-6190790; Department of Ophthalmology, China-Japan Union Hospital of Jilin University 126 Xiantai Street Changchun 130000 China; University of Chinese Academy of Sciences Beijing 100049 China; Department of Environmental Engineering, Technical University of Denmark Kgs. Lyngby DK-2800 Denmark

## Abstract

Correction for ‘Impact of physical structure of granular sludge on methanogenesis and methanogenic community structure’ by Xiaofang Pan *et al.*, *RSC Adv.*, 2019, **9**, 29570–29578.

The authors regret that an incorrect grant number was shown for the Innovation Fund Project in the acknowledgements section of the original manuscript. The correct grant number for the Innovation Fund Project is WES&WQGE201901, and the full corrected acknowledgements section is as shown below.

## Acknowledgements

The authors would like to thank the National Key Research and Development Program of China (Contract No. 2018YFD0500202-4), the National Natural Science Foundation of China (Contract No. 51678553, 21876167, 21477122 and 51808525), the Project of the Natural Science Foundation of Fujian Province (Contract No. 2017J05092), the IUE CAS Young Talents Frontier Project (Contract No. IUEQN201501), the Xiamen Science and Technology Project (Contract No. 3502Z20182003), the FY2015 Japanese-China Research Cooperative Program (Contract No. 2016YFE0118000), the Strategic Priority Research Program of the Chinese Academy of Sciences (Grant No. XDA23030301) and Innovation Fund Project (WES&WQGE201901) for their supports for this study. We would like to thank LetPub (http://www.letpub.com) for providing linguistic assistance during the preparation of this manuscript. We are grateful to Genenergy for help with Illumina sequencing raw data processing.

The Royal Society of Chemistry apologises for these errors and any consequent inconvenience to authors and readers.

## Supplementary Material

